# In-Depth Molecular Characterization of Neovascular Membranes Suggests a Role for Hyalocyte-to-Myofibroblast Transdifferentiation in Proliferative Diabetic Retinopathy

**DOI:** 10.3389/fimmu.2021.757607

**Published:** 2021-11-02

**Authors:** Stefaniya Konstantinova Boneva, Julian Wolf, Rozina Ida Hajdú, Gabriele Prinz, Henrike Salié, Anja Schlecht, Saskia Killmer, Yannik Laich, Henrik Faatz, Albrecht Lommatzsch, Martin Busch, Felicitas Bucher, Andreas Stahl, Daniel Böhringer, Bertram Bengsch, Günther Schlunck, Hansjürgen Agostini, Clemens A. K. Lange

**Affiliations:** ^1^ Eye Center, Medical Center, Faculty of Medicine, University Medical Center Freiburg, Freiburg, Germany; ^2^ Department of Ophthalmology, Semmelweis University, Budapest, Hungary; ^3^ Department of Medicine II, Gastroenterology, Hepatology, Endocrinology and Infectious Disease, Faculty of Medicine, University Medical Center Freiburg, Freiburg, Germany; ^4^ Institute for Anatomy and Cell Biology, Julius-Maximilians-University Würzburg, Würzburg, Germany; ^5^ St. Franziskus Eye Center, Münster, Germany; ^6^ Department of Ophthalmology, University Medical Center Greifswald, Greifswald, Germany

**Keywords:** retinal neovascularization (RNV), proliferative diabetic retinopathy (PDR), transdifferentiation, hyalocytes, myofibroblasts, RNA sequencing, Imaging Mass Cytometry

## Abstract

**Background:**

Retinal neovascularization (RNV) membranes can lead to a tractional retinal detachment, the primary reason for severe vision loss in end-stage disease proliferative diabetic retinopathy (PDR). The aim of this study was to characterize the molecular, cellular and immunological features of RNV in order to unravel potential novel drug treatments for PDR.

**Methods:**

A total of 43 patients undergoing vitrectomy for PDR, macular pucker or macular hole (control patients) were included in this study. The surgically removed RNV and epiretinal membranes were analyzed by RNA sequencing, single-cell based Imaging Mass Cytometry and conventional immunohistochemistry. Immune cells of the vitreous body, also known as hyalocytes, were isolated from patients with PDR by flow cytometry, cultivated and characterized by immunohistochemistry. A bioinformatical drug repurposing approach was applied in order to identify novel potential drug options for end-stage diabetic retinopathy disease.

**Results:**

The in-depth transcriptional and single-cell protein analysis of diabetic RNV tissue samples revealed an accumulation of endothelial cells, macrophages and myofibroblasts as well as an abundance of secreted ECM proteins such as SPARC, FN1 and several types of collagen in RNV tissue. The immunohistochemical staining of cultivated vitreal hyalocytes from patients with PDR showed that hyalocytes express α-SMA (alpha-smooth muscle actin), a classic myofibroblast marker. According to our drug repurposing analysis, imatinib emerged as a potential immunomodulatory drug option for future treatment of PDR.

**Conclusion:**

This study delivers the first in-depth transcriptional and single-cell proteomic characterization of RNV tissue samples. Our data suggest an important role of hyalocyte-to-myofibroblast transdifferentiation in the pathogenesis of diabetic vitreoretinal disease and their modulation as a novel possible clinical approach.

## Introduction

Diabetic retinopathy (DR) is the most common cause of blindness in the working-age population (1) and, given its increasing incidence, it poses a mounting medical and socio-economic challenge. The end-stage disease, proliferative diabetic retinopathy (PDR), is characterized by poorly perfused retinal areas that lead to an uncontrolled release of proangiogenic cytokines *via* a hypoxia-mediated sequence of events, eventually promoting the formation of retinal neovascularization (RNV) growing into the preretinal vitreous (2, 3). Despite great improvements in vitreoretinal surgery in recent years, RNV and resulting tractional retinal detachment still comprise the primary reason for severe vision loss in PDR (4). The vascular endothelial growth factor (VEGF) is so far the only proangiogenic factor directly inhibited in routine clinical practice for the treatment of diabetic macular edema (DME) and PDR by the application of VEGF inhibitors (5). However, since the disease can progress despite continuous anti-VEGF therapy, as evident by the results of the DRCR (Diabetic Retinopathy Clinical Research) Protocol S clinical trial, a contribution of other cellular and molecular mediators in the pathogenesis of PDR is likely (6).

Increasing evidence, including the fact that florid neovascularization growth is attenuated after posterior vitreous detachment (7) and that RNV generally do not recur following vitrectomy (8), points to an important role of the vitreous in the pathogenesis of PDR. However, the scaffold provided by the cortical vitreous does not seem to be the only factor facilitating RNV formation. Vitreous hyalocytes, which represent a unique resident myeloid cell population (9), have been suggested as essential participants in the course of cicatrical contraction in proliferative vitreoretinal disease in the past (10). Nevertheless, the exact role of vitreous hyalocytes in the pathophysiology of RNV in PDR is largely unknown.

To date, most of the studies investigating the cellular components of human RNV have used immunohistochemical approaches and described the presence of CD31-positive endothelial cells, glial fibrillary acidic protein (GFAP)-positive astrocytes and glial cells and α-smooth muscle actin (SMA)-positive myofibroblasts (11, 12). In particular, myofibroblasts represent a unique fibroblast-like cell population with contractile properties in RNV (13) that is not naturally present in the healthy eye, but can be observed in wound healing processes throughout the body (14). However, the cellular origin and contribution of myofibroblasts in RNV in the preretinal vitreous remains the subject of debate.

The aim of this study is to further characterize molecular and cellular features of RNV in order to identify novel drug targets for the treatment of RNV in PDR. To this end, we apply in-depth transcriptional and single-cell protein analyses of human RNV tissue, complemented by *in vitro* studies and *in silico* drug repurposing approaches. We show that RNV are characterized by an accumulation of endothelial cells, M2 macrophages and myofibroblasts and by the presence of a variety of interacting pro-angiogenic, inflammatory and pro-fibrotic factors. Furthermore, our data indicate that vitreal hyalocytes have the potential for myofibroblastic transdifferentiation and thus are involved in the development of RNV in PDR, which may pave the way for new potential immunomodulatory therapeutic approaches.

## Materials And Methods

### Patients’ Characteristics

A total of 43 patients were included in this study (**Supplementary Table 1**). For RNA sequencing analysis, seven retinal neovascular membranes (RNV) from seven patients with PDR, 10 epiretinal membranes (ERM) from 10 patients with idiopathic macular pucker (MP) and seven internal limiting membranes (ILM) from seven patients with idiopathic macular hole (MH) were studied. Two RNV tissue samples from two patients with PDR and four ILM tissue samples from two patients with MP and two patients with MH were analyzed by conventional immunohistochemistry, while five RNV from five patients with PDR and three ERM from three patients with MP were analyzed by Imaging Mass Cytometry. For cultivation of hyalocytes vitreous tissue samples from five patients undergoing vitrectomy for PDR were FACS sorted as previously described (9). All included patients underwent vitrectomy for the respective underlying condition between 2019 and 2021. Relevant previous treatment was recorded prior to surgery. Only patients without concurrent vitreoretinal disease were included (**Supplementary Table 1**). Ethics approval was granted by the local Ethics Committees and a written informed consent was obtained from each patient before tissue acquisition.

### Total RNA Extraction

Tissue samples were immediately stored in RNAlater (Qiagen) at 2-8° until sequencing was performed. RNA extraction, library preparation and RNA sequencing were conducted at the Genomics Core Facility “KFB - Center of Excellence for Fluorescent Bioanalytics” (University of Regensburg, Germany; www.kfb-regensburg.de) as previously described (15). In brief, total RNA was extracted from RNV, ERM and ILM tissue samples and stabilized in RNAprotect Cell Reagent according to the RNeasy Plus Micro Kit protocol (Qiagen). After pelleting, the RNAprotect buffer was removed, replaced by RLT Plus buffer and the samples were homogenized by vortexing for 30 sec. Genomic DNA contamination was eliminated using gDNA Eliminator spin columns. Next, ethanol was added and the samples were applied to RNeasy MinElute spin columns followed by several wash steps. Finally, total RNA was eluted in 12 μl of nuclease-free water.

### RNA-Seq Libraries

RNA sequencing analysis was performed in 24 samples. The SMARTer Ultra Low Input RNA Kit for Sequencing v4 (Clontech Laboratories) was used to generate first-strand cDNA from 750 pg total-RNA. Double-stranded cDNA was amplified by LD PCR (12 cycles) and purified *via* magnetic bead clean-up. Library preparation was carried out as described in the Illumina Nextera XT Sample Preparation Guide (Illumina). 150 pg of input cDNA was tagmented (tagged and fragmented) by the Nextera XT transposome. The products were purified and amplified *via* a limited-cycle PCR program to generate multiplexed sequencing libraries. For the PCR step 1:5 dilutions of index 1 (i7) and index 2 (i5) primers were used. The libraries were quantified using the KAPA SYBR FAST ABI Prism Library Quantification Kit (Kapa Biosystems). Equimolar amounts of each library were pooled, and the pools then used for cluster generation on the cBot with the Illumina TruSeq SR Cluster Kit v3. The sequencing run was performed on a HiSeq 1000 instrument using the indexed, 50 cycles single-read (SR) protocol and TruSeq SBS v3 Reagents according to the Illumina HiSeq 1000 System User Guide. Image analysis and base calling resulted in bcl files, which were converted into fastq files with the bcl2fastq v2.18 software. The sequencing data are available in the Gene Expression Omnibus database under accession number GSE179568.

### Bioinformatics

#### Transcriptional Analysis

Sequencing data was analyzed on the Galaxy web platform (usegalaxy.eu) (16) as previously described (17). Quality control was performed with *FastQC* (*Galaxy Version 0.72*, http://www.bioinformatics.babraham.ac.uk/projects/fastqc/, last access on 8^th^ October 2020). Reads were mapped to the human reference genome (Gencode, release 35, https://www.gencodegenes.org/human/releases.html) with *RNA STAR* (*Galaxy Version 2.7.5b*, default parameters) (18) using the Gencode annotation file (Gencode, Release 35). Three BAM files for each sample (one for each lane) were combined in one BAM file per sample using *Merge BAM files* (*Galaxy Version 1.2.0*). Reads mapped to the human reference genome were counted by *featureCounts* (*Galaxy Version 1.6.4*, default parameters) (19) using the aforementioned annotation file. The output of *featureCounts* was imported to RStudio (version 1.4.1103, R Version 4.0.3). Genesymbols and genetypes were determined based on Ensembl (Release 101, download: 28.10.2020) (20). Genes with 0 reads in all samples were removed from further analysis. Principal component analysis (PCA) (21) was applied to check for potential batch effects. Normalized reads and differential gene expression were calculated using the R package DESeq2 (version 1.30.1) with default parameters [Benjamini-Hochberg adjusted *p*-values (21)]. Transcripts with log2 fold change (log2FC) >2 or <-2 and adjusted *p*-value <0.05 were considered as differentially expressed genes (DEG). Data visualization with read plots was performed using the *ggplot2* R package (22). Heatmaps were created with the R package *ComplexHeatmap* (version 2.6.2) (23). Gene enrichment analysis was performed using the R package *clusterProfiler* (version 3.18.1) (24). Gene ontology (GO) analysis for clusters related to biological processes or molecular functions was performed based on the upregulated genes in RNV using the R function enrichGO of the *clusterProfiler* package with default parameters. Genes associated with the six most disease-relevant biological processes or molecular functions were illustrated using the R function cnetplot of the *clusterProfiler* package with default parameters.

#### xCell Enrichment Analysis

To elucidate the cellular composition of the analyzed tissues we applied the computational method xCell, which quantifies the abundance of 64 immune and stroma cell types including hematopoietic progenitors, epithelial cells, extracellular matrix cells, as well as innate and adaptive immune cells on the basis of transcriptomic data (25). For this purpose, transcripts per million were calculated based on the output of *featureCounts* (assigned reads and feature length) as previously described (26). Enrichment scores were compared between different groups using the Mann-Whitney U test. Cell types with *p <*0.05 were considered to be significantly enriched.

#### Drug Repurposing

Computational screening for drug discovery was performed on the basis of previously published methods (27, 28) using the Connectivity Map (CMap) database (29). A *Gene Set Enrichment Analysis* (GSEA) of genes downregulated in the CMap transcriptional profiles of drug-treated cells and of genes upregulated in RNV according to our analysis was conducted. Results with a significant (adjusted *p* value <0.01) and positive enrichment score were considered for further analysis. Among all significantly enriched drugs, the most relevant ones were identified by analyzing their modulating effect on, according to our GO enrichment analysis, four RNV-associated processes (“*angiogenesis*”, “*leukocyte migration*”, “*adaptive immunity*” and “*extracellular structure organization*”). For every drug in question the overlap of upregulated DEG in RNV and genes downregulated in cells treated with the respective drug was determined to predict modulated genes in RNV. In a next step, the percentage of overlapping genes associated with the disease-relevant biological processes listed above was calculated. This information, together with the enrichment score of the GSEA analysis, was used to prioritize drugs. The sum of enrichment score and mean of percentage of overlapping genes associated with the four biological processes was calculated to rank drugs according to their relevance.

### Standard Immunohistochemistry

RNV and ILM tissue samples were processed for conventional immunohistochemical analysis. The membranes were collected in isotonic electrolyte solution after surgical removal, immediately transferred to 4% paraformaldehyde (PFA) diluted in 0.027 M PBS (phosphate-buffered saline, pH = 6.7-6.8) and placed on ice for 1 hour. Following extensive rinsing with PBS, the samples were incubated for at least 4 hours at 4°C in 20% sucrose diluted in 0.027 M PBS prior to embedding in Tissue-Tek O.C.T. embedding medium (Sakura). 10-μm-thick cryosections were cut and stored at −20°C until use. All sections were blocked in a solution of 5% normal donkey serum (Jackson ImmunoResearch) and 1% bovine serum albumin (BSA) (Roth) in 0.027 M PBS Triton X-100 0.3% (Sigma-Aldrich) for 1 hour at room temperature. Immunostaining was performed by applying primary antibodies against Cluster of Differentiation 206 (CD206, 1:2500, abcam, Cat#ab64693), ionized calcium-binding adaptor molecule 1 (IBA-1, 1:500, abcam, Cat#ab5076), fibronectin (FN1, 1:500, Sigma-Aldrich, Cat#F6140) and secreted protein acidic and rich in cysteine (SPARC, 1:500, Sigma-Aldrich, Cat#HPA002989) overnight at 4°C. Primary antibodies were diluted in the same blocking solution and were omitted for negative control. Following extensive rinsing in PBS, sections were stained with an Alexa Fluor^®^ 568-coupled donkey anti-goat, an Alexa 488-coupled donkey anti-mouse or a Cyanine Cy™5-conjugated donkey anti-rabbit secondary antibody (diluted 1:500 in the blocking buffer) at room temperature for 1 hour. After repeated rinsing with 0.027 M PBS, sections were counterstained for 10 minutes with 4′,6-Diamidin-2-phenylindol (DAPI) (Sigma-Aldrich) diluted 1:1000 in 0.027 M PBS and rinsed again in 0.027 M PBS three times. Finally, autofluorescence quenching was performed with TrueBlack Lipofuscin Autofluorescence Quencher (Biotium) according to the manufacturer’s protocol. Representative images were taken on a Leica TCS SP8 Confocal System coupled to a Leica DMi8 inverted microscope equipped with 20x (0.75 NA) and 40x (0.95 NA) air objectives.

### Imaging Mass Cytometry

#### Tissue Preparation

For Imaging Mass Cytometry five RNV tissue samples and three ERM were processed as previously described (30, 31). In brief, samples were fixed in 4% formalin for 12 hours immediately after resection and subsequently dehydrated. After incubation in xylene, the samples were placed in liquid paraffin for 4 hours and afterwards embedded. For staining, 6-µm-thick sections were made and mounted on slides. Prior to staining, paraffin slides were deparaffinized in xylene and rehydrated. Heat-induced antigen retrieval was performed using DAKO EnvisionFlex target retrieval solution (high pH, Agilent Technologies) at 95°C for 30 min in a pressure cooker. Subsequently, slides were blocked in 3% BSA in tris-buffered saline (TBS) and a specifically compiled panel of antibodies (Fluidigm) was used to stain the sections. A complete list of antibodies, clones and conjugated metals used in this study is shown in **Supplementary Table 2**. 1:100 (VEGF, Arginase 1) or 1:800 (other antibodies) diluted antibodies were applied to sections simultaneously within an antibody mix and incubated overnight at 4°C in a hydration chamber. After washing with TBS, an iridium-intercalator solution (1:2000 in TBS) was applied to the slides. Finally, the sections were subjected to laser ablation and data acquisition.

#### Image Acquisition

Images were acquired using the Hyperion Imaging System (Fluidigm). Tuning of the instrument was performed according to manufacturer’s instructions. Regions of interest were determined by dark-field microscopy before acquisition. Tissue sections were laser-ablated spot-by-spot at 200 Hz resulting in a pixel size/resolution of 1 µm². Several 1500 µm² images per sample were produced. Raw data was processed using the CyTOF software v7.0 (Fluidigm). Images were controlled on the MCD Viewer v1.0.560.6 (Fluidigm).

#### Segmentation and High-Dimensional Data Analysis

Imaging Mass Cytometry (IMC) data was processed as previously described (32). In brief, mcd files generated in the process of data acquisition were converted into tiff image stacks following a Python script adapted from https://github.com/BodenmillerGroup/ImcSegmentationPipeline. Hereafter, segmentation masks were developed by using the *ilastik* software (33) (version 1.3.2) to designate nuclei, cytoplasm and background fractions and subsequently uploading the probability maps into *CellProfiler* (34) (Version 3.1.8) to create cell masks, which were used to extract single-cell information. Resulting data folders were uploaded into *histoCat* (35) (Version 1.76) to calculate mean marker intensity of pixels and data was normalized to the 99^th^ percentile for PhenoGraph clustering (36) (nearest neighbors = 15). Clustering was performed based on data from the channels showing a plausible staining: α-SMA, Vimentin, CD16, CD163, CD31 (PECAM-1), CD45, CD44, β-Actin, CD68, CD8a, VEGF, CD74, Ki-67, Collagen Type I, Histone H3, CD276, HLA-DR, Pan-Actin, Nucleic acid. Single-cell cluster data was exported for further analysis to Omiq.ai (Omiq). *opt*-SNE (optimized t-Distributed Stochastic Neighbor Embedding) dimensionality reduction (37) was used to visualize the cellular profile on a single-cell basis with the following settings: arcsinh transformation cofactor: 0.2, max iterations: 1000, *opt*-SNE end: 5000, perplexity: 30, theta: 0.5, random seed: 2549, verbosity: 25. Marker expression in distinct Phenograph clusters was calculated in Omiq and visualized as a heatmap using *ComplexHeatmap 1.20.0* (23) in RStudio (Version 1.4.1103, R Version 4.0.3). Barplots showing cluster assembly between entities were created using the *ggplot2* package (22). Statistical analysis was performed in GraphPad Prism (GraphPad Software, version 6.0). Differences in cell population counts were analyzed by a *t*-test with Welch’s correction.

### Fluorescence-Activated Cell Sorting (FACS)

Vitreous tissue samples from five patients with PDR were collected in vitrectomy bags, immediately placed on ice in the operating theatre and processed for cell isolation within two hours of surgical resection as previously described (9). Briefly, following centrifugation the vitreous pellet was digested with Collagenase D (5 mg/mL, Roche) and DNase I (1 mg/mL, Roche) in HBSS (Hanks’ balanced salt solution) for 20 min at 37°C. 1 mL of Red Blood Cells (RBC) Lysis Buffer (Thermo Fisher Scientific) was added to each FACS tube for erythrocyte lysis after removing the supernatant. Following another centrifugation step (7 min at 250x g at RT) 0.5 µL of Fixable Viability Dye (eFluor 780, eBioscience) per 1 mL of cells was added. The pellet was stained for CD45 (BV421, anti-human, 1:100, BioLegend), CD11b (FITC, anti-human, 1:100, BioLegend), CX_3_CR1 (PE-Cy7, anti-human, 1:200, BioLegend) and Anti-Human Mature Macrophages (MatMac) Antibody (eFluor660, anti-human, 1:100, eBioscience). Finally, cells were re-suspended in FACS buffer and processed for sorting on the MoFlo Astrios EQ Cytometer (Beckman Coulter).

### Cultivation and Staining of Sorted Hyalocytes

Sorted cells were transferred to collagen-coated chamber slides (PureCol Type I Collagen Solution, 3.1 mg/ml, diluted 1:1000 in PBS, Advanced BioMatrix), the hyalocyte medium containing RPMI 1640 (PAN), 10% FCS (Fetal Calf Serum), 1 mM sodium pyruvate (Gibco), 10 mM HEPES (4-(2-hydroxyethyl)-1-piperazineethanesulfonic acid, PAN), Penicillin-Streptomycin (Sigma-Aldrich) and 20 ng/ml M-CSF (macrophage colony-stimulating factor, R&D Systems), and kept in culture for five to 12 days. Fixation with PFA 2% was performed prior to an overnight blocking with 1% BSA and 5% donkey serum in PBS Triton X-100 0.1% (Gibco). Subsequently, cells were incubated with IBA-1 (diluted 1:500, abcam, Cat#ab5076) and α-SMA (1:500, Sigma-Aldrich, Cat#2547-100UL) primary antibodies overnight. Incubation with secondary antibodies (Alexa 647 (1:500) and Alexa 488 (1:500) from Thermo Fisher Scientific) and Phalloidin TRITC (5 (6)-Tetramethylrhodaminisothiocyanat, diluted 1:500, Sigma-Aldrich) was performed for one hour. Nuclei were counterstained with DAPI (1:1000) and the specimen were embedded in Fluorescence Mounting Medium (Agilent Dako). Representative images were taken on a Leica TCS SP8 Confocal System coupled to a Leica DMi8 inverted microscope equipped with 20x (0.75 NA) and 40x (0.95 NA) air objectives.

In order to verify the staining pattern of cultured hyalocytes under more physiological condition, we plated hyalocytes obtained during vitrectomy from one more patient with PDR on a collagen-coated (10 µg/ml) 0.25% polyacrylamide gel (PA). The custom-made PA hydrogels were crosslinked with 0.2% (final concentration) bis-acrylamide following procedures published earlier (38, 39). This cultivation method has been described to result in a significant loss of α-SMA expression in trabecular meshwork cells plated on 0.2% bis-acrylamide gels as compared to stiffer gels or tissue culture plastic (39), strongly suggesting that there is a lower tendency to induce α-SMA on a 0.2% bis-acrylamide PA gel substrate. Hyalocytes cultured in PA hydrogels were stained and imaged, as described above.

## Results

### Patients’ Characteristics

**Figure 1:**
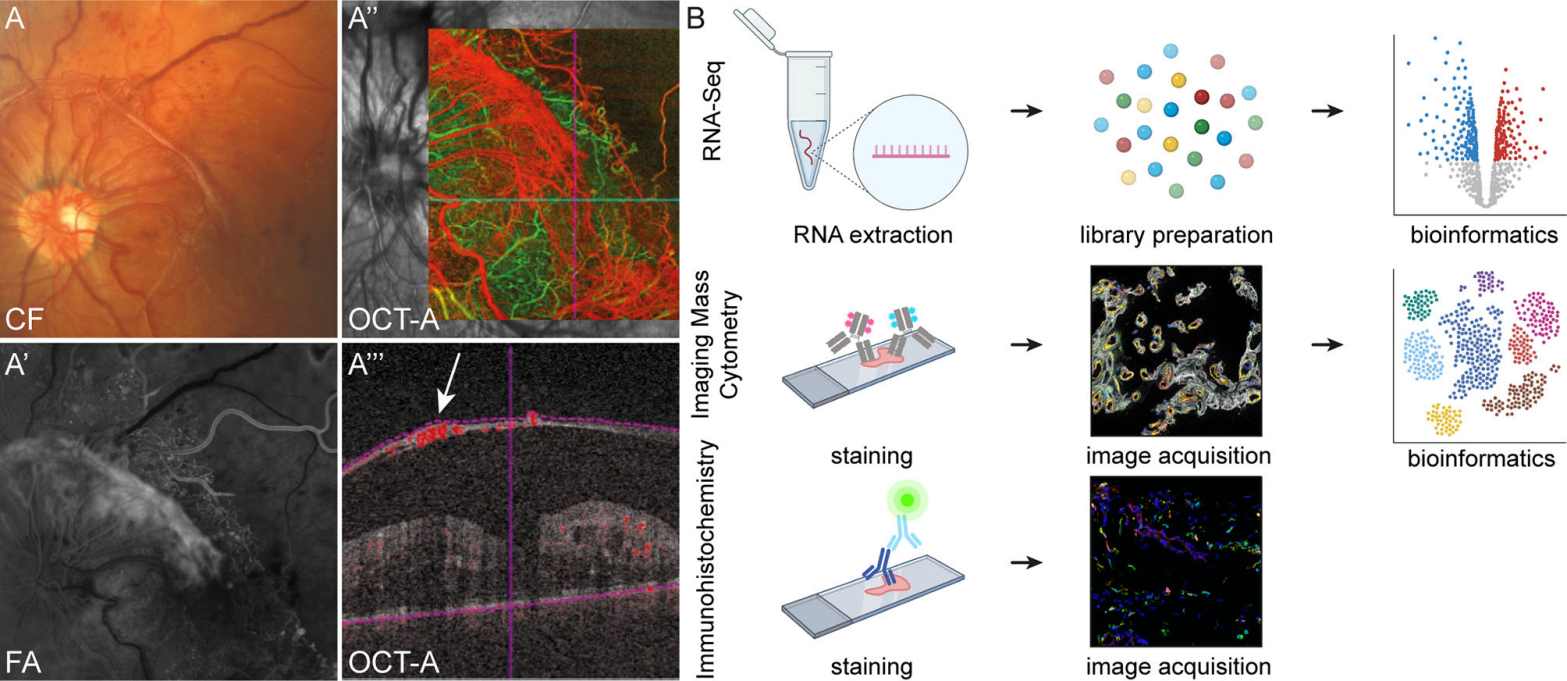
Experimental setup. Retinal neovascularization membranes (RNV) were visualized prior to surgery *via* funduscopic examination **(A)** color fundus, CF), fluorescein angiography, FA (A’) and optical coherence tomography angiography, OCT-A (A’’ and A’’’). A’’’ The arrow points to a preretinal membrane interspersed with blood vessels. **(B)** Workflow for tissue processing. RNV and control tissue samples (epiretinal membrane and internal limiting membrane, not shown) for RNA sequencing were stored in RNAlater immediately upon resection before RNA extraction and generation of first-strand cDNA were performed (upper panel). Following library preparation and RNA sequencing, bioinformatic analyses were conducted. For Imaging Mass Cytometry (IMC), excised tissue samples were collected in formalin, processed for paraffin embedding (FFPE, middle panel) and sectioned. For staining of tissue sections, a compilation of 27 metal isotope-conjugated antibodies was applied before image acquisition. In the following, segmentation of IMC data *via* supervised machine learning was conducted prior to high-dimensional data analysis. Conventional immunohistochemistry (IHC, lower panel) was performed on cryo-conserved tissue sections and visualized by confocal microscopy.

A total of 43 patients undergoing vitrectomy for PDR, MP or MH were included in this study (**Supplementary Table 1**). Diagnosis was based on a thorough funduscopic exam, spectral domain optical coherence tomography (OCT), fluorescein angiography (FA, HRA2, Heidelberg Engineering) and OCT-angiography (AngioPlex, Zeiss) (**Figure 1A**). Seven RNV membranes from seven PDR patients (mean age 38 ± 14.8 years), 10 ERM from 10 patients with MP (mean age 71 ± 6.8 years) and seven ILM from seven patients suffering from MH (mean age 68 ± 5.6 years) were processed for RNA sequencing analysis (**Figure 1B**, upper panel). Imaging Mass Cytometry was performed on eight tissue samples [RNV membranes from five PDR patients (mean age 43 ± 12.3 years) and ERM from three MP patients (mean age 69 ± 5.0 years)] (**Figure 1B**, middle panel), while six samples (RNV membranes from two PDR patients [mean age 30.5 ± 2.1 years) and ILM from two MP and two MH patients (mean age 77.8 years)] were stained for conventional immunohistochemical analysis (**Figure 1B**, lower panel). Vitreous tissue samples from five patients undergoing vitrectomy for PDR (mean age 58 ± 21.3 years) were FACS-sorted and processed for cell culture experiments. Patient characteristics are summarized in **Supplementary Table 1**.

### Transcriptomic Cellular Profiling of Diabetic and Non-Diabetic Epiretinal Membranes

**Figure 2:**
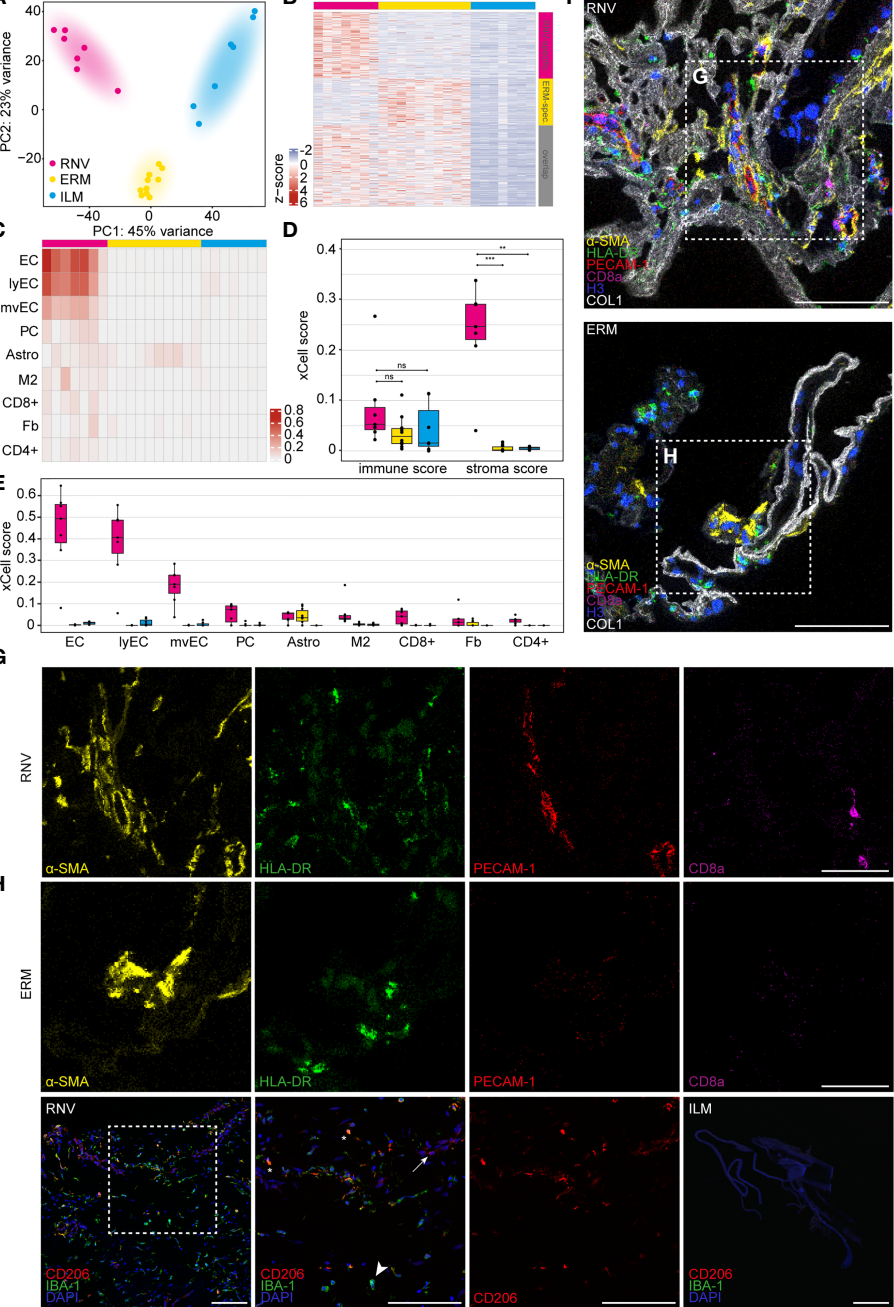
Diabetic retinal neovascularization membranes (RNV) differ significantly in their cellular and molecular composition from epiretinal (ERM) and internal limiting membranes (ILM). **(A)** Principal component analysis (PCA) plot demonstrating the distribution of the three analyzed entities: RNV (magenta), ERM (yellow) and ILM (blue). **(B)** Heatmap of the genes upregulated in RNV (upper magenta bar) and ERM (upper yellow bar) when compared to ILM (upper blue bar). The side bars mark RNV-specific (side magenta bar), ERM-specific (side yellow bar) genes or the overlap between both (grey bar), each in comparison to ILM (log2 fold change >2, adjusted *p* value <0.05). Color coding of the transcripts according to the z-score (deviation from a gene’s mean expression in standard deviation units). **(C)** Heatmap of significantly enriched cell types in RNV (magenta) *vs*. ILM (blue) according to the xCell analysis. Each row represents one cell type, each column one sample. The rows are ordered according to the fold change of the mean enrichment scores between RNV and ILM. EC, endothelial cells. ly, lymphatic. mv, microvascular. PC, pericytes. Astro, astrocytes. M2, M2 macrophages. CD8+, CD8+ central memory T cell. Fb, fibroblasts. CD4+, CD4+ naive T cells. Color coding for cell types. **(D)** Boxplots of the xCell immune and stroma scores in RNV (magenta), ERM (yellow) and ILM (blue). Each dot represents one sample. *p <0.05, **p <0.005, ***p <0.0005. ns, not significant (Mann-Whitney U test). **(E)** Boxplots of significantly enriched cell types in RNV (magenta) *vs*. ILM (blue), ordered according to the log2 fold change between RNV and ILM. **(F)** Imaging Mass Cytometry on RNV and ERM tissue samples. Images are representative for all stained samples. Multiplexed staining for α-SMA (α-smooth muscle actin, yellow), HLA-DR (human leukocyte antigen – DR isotype, green), PECAM-1 (platelet endothelial cell adhesion molecule, red), CD8a (magenta), Histone H3 (blue) and COL1 (collagen type I, white) is presented. Higher magnification of the sections within the dashed white square is shown respectively in the panels in **(G, H)** Scale bars correspond to 100 μm **(F)** and 50 μm **(G, H)**. **(I)** Immunohistochemical staining for CD206 (Cluster of Differentiation 206) and IBA-1 (ionized calcium-binding adapter molecule 1) in RNV and ILM samples. Higher magnification of the section within the dashed white square is shown to the right. Double-positive CD206^+^IBA-1^+^ cells are tagged by an asterisk. Arrow points to a CD206^+^IBA-1^-^ cell. Arrowhead points toward a CD206-IBA-1^+^ cell. Nuclei are counterstained with DAPI. Scale bars correspond to 100 μm.

RNA-Seq of the vitreoretinal tissue yielded a mean total number of 34.5 million raw reads (range 32.3-41.1) in the RNV group, 35.6 million raw reads (range 31.5-43.3) in the ERM group, and 35.8 million raw reads (range 30.7-43.6) in the ILM group. A sufficient amount of reads from each sample could be assigned to the human reference genome, thus all included samples were processed for further bioinformatic analysis. A primary unsupervised assessment of the data in a principal component analysis (PCA) and in a heatmap of the upregulated genes revealed a clear distinction of the three entities on the transcriptional level (**Figures 2A, B**). Genes specific for RNV or ERM were identified based on factors upregulated in comparison to ILM (log2 fold change >2, adjusted *p <*0.05), and deducting the overlap (genes differentially upregulated in both entities when compared to ILM) (**Figure 2B**). According to our analysis, 1517 genes were RNV-specific, 1068 genes were ERM-specific, and 1848 were differentially upregulated in both tissue samples when compared to ILM.

Since the transcriptional signature represents a conglomerate of the RNA of multiple cell types, we next conducted a bioinformatic cell type enrichment analysis to decipher the identity of the cellular populations in RNV, ERM and ILM using the gene expression-based cell type enrichment tool xCell (25). Cell type enrichment scores across 64 immune and stromal cell types were calculated for the three entities. The nine cell types significantly enriched in RNV when compared to ILM are presented in the heatmap in **Figure 2C**. While only a trend to higher mean immune scores was observed in RNV samples, stroma scores representing the stromal cell content were significantly increased in RNV when compared to both ERM and ILM (**Figure 2D**). Across the cell subpopulations, different kinds of endothelial cells and fibroblasts belonged to the most enriched cell populations in RNV. Interestingly, immune cells, such as M2 macrophages and T cells, were also among the nine most enriched cell types in RNV compared to ERM and ILM, indicating a smoldering immune response in RNV from patients with PDR (**Figure 2E**). Next, we investigated whether these findings could be recapitulated on the protein level by assessing the expression of α-SMA, HLA-DR (human leukocyte antigen – DR isotype), PECAM-1 (platelet endothelial cell adhesion molecule), CD8a, histone H3, and collagen type I by Imaging Mass Cytometry analysis (**Figure 2F**). In line with the RNA-Seq results, we observed an abundance of α-SMA^+^ myofibroblasts, HLA-DR^+^ antigen-presenting immune cells, PECAM-1^+^ endothelial cells and CD8a^+^ cytotoxic lymphocytes in RNV (**Figure 2G**) compared to a fainter staining for these markers in ERM (**Figure 2H**). In order to elucidate the nature of accumulating macrophages in RNV, we performed an immunohistochemical co-staining for the macrophage mannose receptor CD206 (Custer of Differentiation 206), a M2 macrophage marker, and IBA-1 (ionized calcium-binding adapter molecule 1), a myeloid cell marker, on RNV and ILM tissue samples (**Figure 2I**). Double-positive CD206^+^IBA-1^+^ were abundant in the RNV samples, confirming the xCell enrichment analysis. However, CD206^-^IBA-1^+^ and CD206^+^IBA-1^-^ were also present in RNV tissue, suggesting the involvement of other myeloid cell populations in the pathogenesis of the disease. ILM samples stained negative for both markers.

### Transcriptional Characterization of Diabetic and Non-Diabetic Epiretinal Membranes

**Figure 3:**
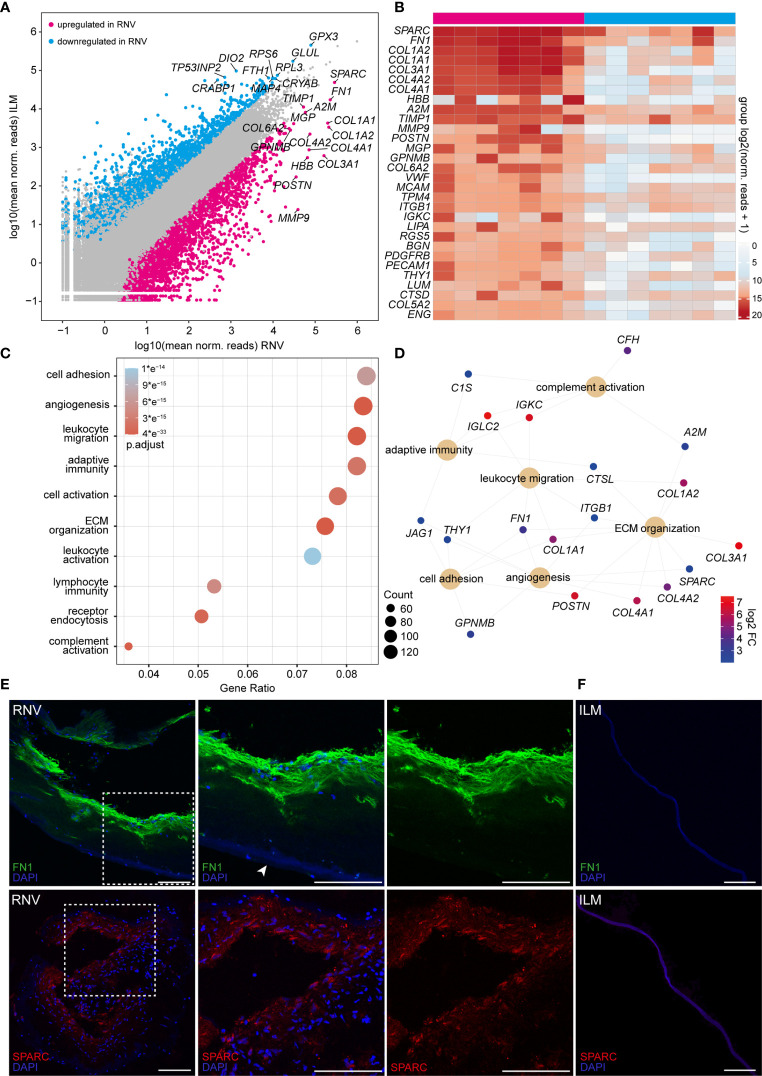
High-throughput sequencing identifies a variety of pro-angiogenic, inflammatory and pro-fibrotic factors enriched in retinal neovascularization (RNV) membranes. **(A)** Readplot of differentially expressed genes in RNV (upregulated genes in magenta, downregulated genes in blue, not differentially expressed genes in grey; the most strongly expressed genes from each group are labeled). **(B)** Heatmap of the most prominent transcripts in human RNV membranes according to the mean expression. Color coding of the transcripts according to the number of normalized counts (log2-scaled). **(C)** Most significant Gene ontology (GO) biological process clusters of the significantly upregulated mRNA transcripts in RNV. Color coding of the dots according to the adjusted *p* value, size of the dots according to the count of transcripts associated with the respective GO term. ECM, extracellular matrix. **(D)** Cnetplot of the top expressed genes in the most disease-relevant GO biological processes. Color coding of the transcripts according to the log 2 fold change of mean expression. **(E)** Immunohistochemical staining for FN1 (upper panel) and SPARC (lower panel) in RNV samples. Higher magnification of the sections within the dashed white square is shown to the right. Arrowhead points to the ILM, adjacent to the RNV. Nuclei are counterstained with DAPI. **(F)** Immunohistochemical staining for FN1 (upper panel) and SPARC (lower panel) in internal limiting membrane (ILM) samples. Nuclei are counterstained with DAPI. Scale bars correspond to 100 μm.

Having established the cellular composition profile of RNV, we next focused on the molecular changes in RNV. To this end, we chose the ILM of MH patients as control tissue, since it serves as a scaffold for cell proliferation in vitreomacular interface disease and is thus the most physiological correlate of RNV accessible by vitreoretinal surgery. Comparative analysis of the transcriptome of RNV and ILM revealed 3365 genes that were differentially upregulated in RNV, whereas 2114 factors were differentially downregulated in RNV (**Figure 3A**). The gene signature of RNV was denoted by the expression of a variety of extracellular matrix (ECM) proteins, such as *SPARC*, *FN1*, several collagen types (*COL1A2*, *COL1A1*, *COL3A2*, *COL4A2*, *COL4A1*, *COL6A2*, *COL5A2*) and classic endothelial cell markers, such as *MCAM* (melanoma cell adhesion molecule), *PDGFRB* (platelet-derived growth factor receptor beta) and *CD31*/*PECAM-1* (**Figure 3B**). Furthermore, several factors associated with the regulation of immune processes, such as *MMP9* (matrix metallopeptidase 9), *A2M* (alpha-2-macroglobulin) and *TIMP-1* (tissue inhibitor of metalloproteinase-1), and genes involved in smooth muscle contraction, such as *TPM4 (*tropomysin alpha-4 chain) and *RGS5* (regulator of G-protein signaling-5), lined up among the 30 most prominent transcripts in human RNV.

To gain more insight into the biological pathways involved in the formation of RNV, we performed a GO cluster analysis of the differentially upregulated genes in RNV (**Figure 3C**). The GO enrichment analysis of biological processes (BP) involved in RNV showed that the differentially upregulated factors in RNV most significantly contribute to “*regulation of cell adhesion*” (GO:0030155, adjusted *p <*6.3e-15), “*angiogenesis*” (GO: 0001525, adjusted *p <*2.1e-21), “*leukocyte migration*” (GO:0050900, adjusted *p <*2.0e-27), “*adaptive immune response*” (GO:0002250, adjusted *p <*2.5e-15) and “*regulation of cell activation*” (GO:0050865, adjusted *p <*1.9e-15) (**Figure 3C**). The GO enrichment analysis of molecular functions (MF) in RNV revealed “*glycosaminoglycan binding*” (GO:0005539, adjusted *p <*1.3e-09), “*growth factor binding*” (GO:0019838, adjusted *p <*2.6e-14) and “*cytokine binding*” (GO:0019955, adjusted *p <*4.2 e-13) as the most enriched GO terms in RNV (**Supplementary Figures 1D, E**). All enriched GO BP and MF terms with corresponding gene count are summarized in **Supplementary Tables 3** and **4**. Looking more closely into two top GO biological processes enriched in RNV, “*angiogenesis*” and “*immune response*”, we found that *SPARC*, *FN1*, *COL4A2* (collagen type IV alpha 2), *COL4A1* (collagen type IV alpha 1 chain), *MCAM*, *PDGFRB* and *PECAM-1*, among others, are important participants in the “*angiogenesis*” process, while *A2M*, *MMP9*, *ITGB1* (integrin subunit beta 1) and *CTSD* (cathepsin D) contribute to the enrichment of “*immune response*” in RNV (**Supplementary Figures 1A, B**). Interestingly, *FN1* emerged as one of the most prominent factors linking four BP clusters enriched in RNV, including “*cell adhesion*”, “*angiogenesis*”, “*leukocyte migration*” and “*ECM organization*” (**Figure 3D**). *SPARC*, on the other hand, stood out as the highest expressed gene within the “*angiogenesis*” term that also contributes to “*ECM organization*” (**Figure 3D** and **Supplementary Figure 1C**). To validate the expression of these two central factors on the protein level, we next performed immunohistochemistry against FN1 and SPARC on RNV and ILM tissue samples. Here, we found a strong immunoreactivity for FN1 and SPARC in the RNV membranes of patients with PDR compared to an only faint staining in the ILM of patients with MP (**Figures 3E, F**), supporting our RNA-Seq analysis results and suggesting an essential role of these two factors in the development of RNV. Negative controls, omitting the primary antibodies, and autofluorescence controls are provided in **Supplementary Figure 2**.

### Imaging Mass Cytometry (IMC) of RNV and ERM

**Figure 4:**
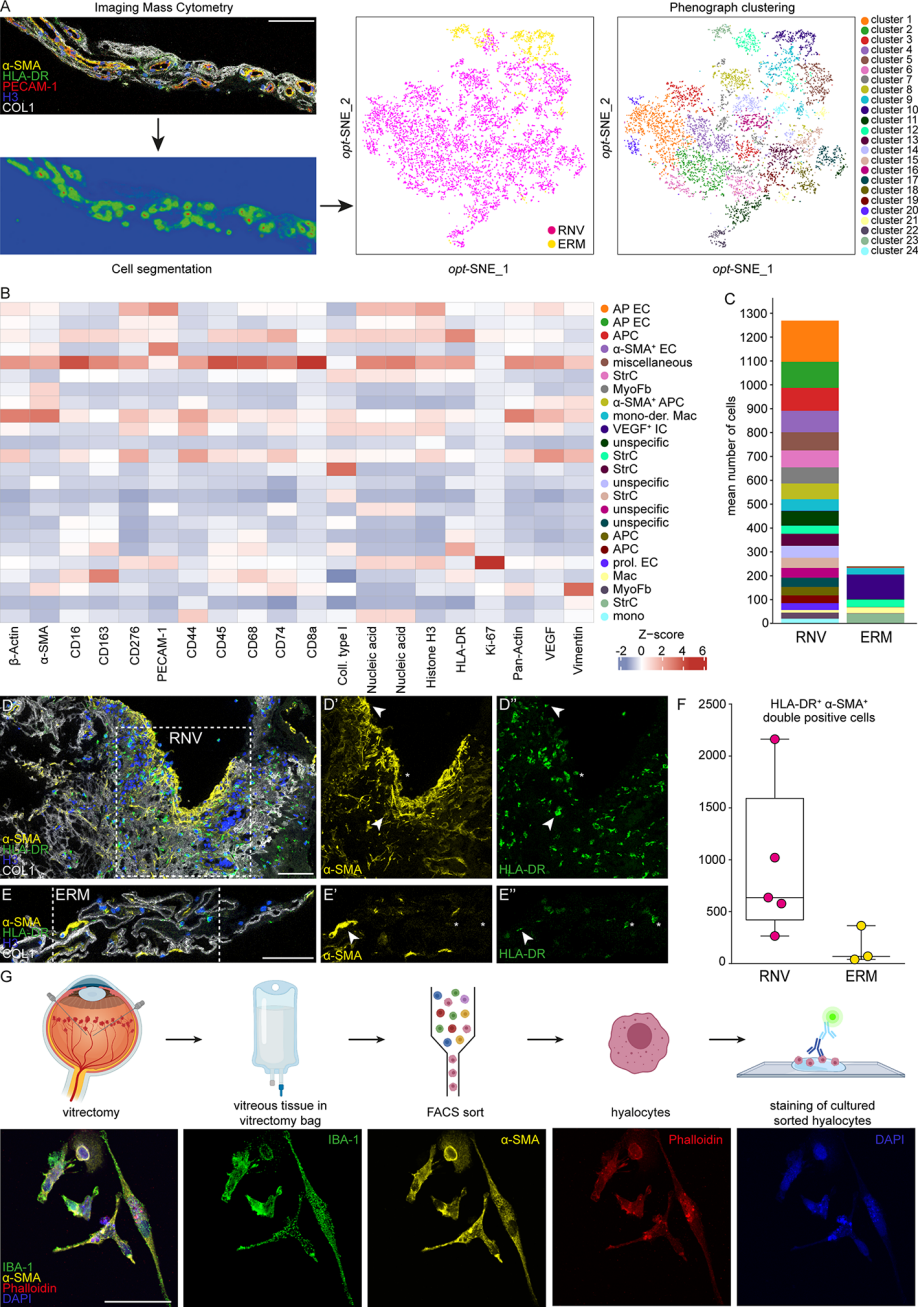
Single-cell protein analysis of retinal neovascularization (RNV) reveals a pro-fibrotic inflammatory phenotype. **(A)** Highly multiplexed Imaging Mass Cytometry data was segmented into cellular masks *via* supervised machine learning. Finally, single-cell data was extracted and cells were attributed to 24 distinct clusters using Phenograph on an *opt*-SNE map. The data were further plotted to the cellular origin (RNV samples in magenta and epiretinal membrane (ERM) samples in yellow). **(B)** Heatmap of marker signal intensity in the Phenograph clusters depicted in A. Z-score: deviation from a marker’s mean expression in standard deviation units. Annotation of clusters was performed according to specific marker expression. AP EC, antigen-presenting endothelial cells. APC, antigen-presenting cells. α-SMA^+^ EC, α-smooth muscle actin positive endothelial cells. StrC, stroma cells. MyoFb, myofibroblasts. mono-der. Mac, monocyte-derived macrophages. VEGF^+^ IC, vascular endothelial growth factor positive immune cells. prol. EC, proliferating endothelial cells. **(C)** Cluster assembly was compared between RNV and ERM and visualized in stacked bar charts displaying mean counts per group. Multiplexed staining for HLA-DR (human leukocyte antigen – DR isotype, green), α-SMA (α-smooth muscle actin, yellow), Histone H3 (blue) and COL1 (collagen type I, white) was performed on RNV **(D)** and ERM **(E)** samples. The single stainings (α-SMA and HLA-DR) for the section within the dashed white square are shown in the panels to the right. Arrowheads point to double-positive HLA-DR^+^α-SMA^+^ cells. Asterisks indicate α-SMA^-^HLA-DR^+^ cells. Scale bars correspond to 100 μm. **(F)** Absolute cell counts of double-positive HLA-DR^+^α-SMA^+^ cells were compared between RNV (magenta) and ERM (yellow) tissue samples. Data are presented as box-and-whiskers plots. Each dot represents one sample. **(G)** Upper panel: schematic illustrating the workflow for cultivation and staining of human hyalocytes. Lower panel: immunohistochemical staining of hyalocytes from a PDR patient for IBA-1 (green), α-SMA (yellow) and Phalloidin (red). Nuclei are counterstained with DAPI (4′,6-Diamidin-2-phenylindol). Scale bars correspond to 50 μm.

To further validate our RNA-Seq analysis on the protein level and to gain insight into the cellular components of RNV, we next performed IMC on RNV and ERM tissue. Since the ILM is mostly acellular and thus not suitable for IMC, we employed ERM as control tissue in this series of experiments. IMC allows to assess the localization of multiple proteins in parallel since the highly specific isotope-based primary antibody detection avoids the limitations of common fluorophore-based systems. Using this technique, we simultaneously studied the distribution of 18 proteins in RNV and ERM tissue (**Supplementary Figure 3**). After image acquisition, supervised machine learning was harnessed for image segmentation following a previously published protocol (32) (**Figure 4A**). By Phenograph clustering 24 distinct cell clusters were distinguished in RNV and ERM tissue based on the high-dimensional single-cell IMC data. 21 clusters could be assigned to RNV, while only three clusters (cluster 10, cluster 21 and cluster 23) could be clearly attributed to ERM (**Figure 4A**). The most enriched clusters in RNV were annotated according to their expression profile as antigen-presenting endothelial cells, antigen-presenting cells, α-SMA^+^ endothelial and antigen-presenting cells, stroma cells and myofibroblasts (**Figures 4B, C**). Interestingly, IMC revealed a considerable number of HLA-DR^+^ immune cells, which were also positive for α-SMA in RNV when compared to ERM (*p*=0.07) (**Figures 4D–F**). The noticeable concomitant expression of α-SMA in HLA-DR^+^ immune cells in RNV prompted us to explore the origin of these myeloid cells with myofibroblastic features in more detail.

### Cultivation of Sorted Vitreal Hyalocytes From Patients With Proliferative Diabetic Retinopathy

Human hyalocytes represent the resident myeloid cell population in the vitreous and mainly occupy the posterior vitreous cortex adjacent to the inner retinal surface, the site of RNV development. Since hyalocytes express HLA-DR (9), accumulate in RNV (15, 40) and are accessible through vitreoretinal surgery, we next isolated human hyalocytes from the vitreous of four patients with PDR and cultured the cells in four independent experiments according to a protocol specifically designed for this study (**Figure 4G**, upper panel). Immunohistochemical staining revealed a strong expression of IBA-1 in hyalocytes confirming their myeloid cell origin. Interestingly, and in line with the above-mentioned IMC results, we observed an enhanced immunofluorescent signal for α-SMA in hyalocytes, which indicates a myofibroblastic phenotype (**Figure 4G**, lower panel). These data strongly suggest that vitreal hyalocytes are capable of transdifferentiating into myofibroblasts during the course of severe proliferative diabetic retinopathy and may thus contribute to disease progression.

In order to verify the staining pattern of cultured hyalocytes under more physiological conditions, we plated hyalocytes obtained during vitrectomy for PDR from one more patient on a collagen-coated polyacrylamide gel, thus minimizing the biomechanical impact of the system. We observed a similar staining pattern as in our initial experiments on stiff slides, revealing a strong expression of IBA-1 and α-SMA in hyalocytes (**Supplementary Figure 4**). This additional experiment under more physiological conditions emphasizes the potential for myofibroblastic transdifferentiation of hyalocytes.

### Computational Screening for Drug Discovery

**Figure 5:**
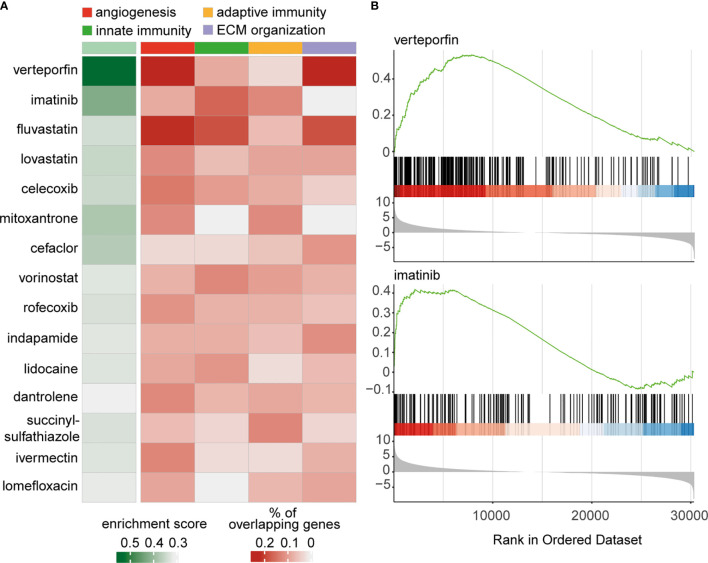
Transcriptome-based drug repurposing for application in retinal neovascularization. **(A)** Heatmap of drugs for potential application in treatment of retinal neovascularization. The enrichment score was calculated for drugs, associated with the modulation of four disease-relevant processes: “*angiogenesis*” (red), “*innate immunity*” (green), “*adaptive immunity*” (ocher) and “*extracellular structure organization*” (lilac). The drugs are ordered according to the sum of enrichment score and mean of percentage of overlapping genes associated with the four biological processes. **(B)** *Gene set enrichment analysis* (GSEA) enrichment score curves for the most prominent drugs in above-mentioned analysis – verteporfin and imatinib. The green curves in the upper part of both graphs depict the enrichment score, which is the running sum of the weighted enrichment score for the respective drug. The lower parts show the ranked list of RNV genes ordered by llog2 fold change (RNV *vs*. ILM); every gene, which is downregulated by the indicated drug, is represented by a black bar in the middle of the plots.

Although panretinal photocoagulation and, more recently, anti-VEGF therapy are effective treatments for RNV, these strategies are only successful in 60% (41) and 70% (42) of cases, respectively. This highlights the need to explore further therapeutic options for the treatment of RNV in PDR. In order to screen for drugs, which could influence disease progression, we applied a transcriptome-based drug-repurposing approach. In brief, we used drug-exposure transcriptome data from the CMAP database (29) as a reference and screened a variety of drug-induced transcriptional profiles in relation to our RNV signature using GSEA. Hereby, a score reflecting the significant enrichment of genes upregulated in RNV and downregulated in the CMap transcriptional profiles of drug-treated cells was calculated for each drug (**Figure 5A**). Drugs were considered most relevant, when the *in silico* analysis predicted modulation of disease-relevant processes such as “*angiogenesis*”, “*leukocyte migration*”, “*adaptive immunity*” and “*extracellular structure organization*”. The overlap of upregulated DEG in RNV and genes downregulated in cells treated with the respective drug was determined to predict genes in RNV, which might be therapeutically modulated by the respective drug. The sum of enrichment score and mean percentage of overlapping genes associated with the four biological processes was calculated to rank drugs according to their potential relevance, as shown in **Figure 5A** (refer to *Materials and Methods* for details). According to this analysis, the benzoporphyrin derivate verteporfin, known as a photosensitizer applied in photodynamic therapy for wet macular degeneration, myopic choroidal neovascularization and central serous chorioretinopathy, was the most disease-relevant drug for treatment of RNV (**Figure 5B**). Imatinib, a 2-phenyl amino pyrimidine derivate, which lined up right after verteporfin, acts as a specific inhibitor of tyrosine kinases (**Figure 5B**).

## Discussion

In light of the increasing number of patients with diabetic retinopathy, which is expected to nearly triple by 2050 (43), research into effective treatment options, particularly for the visual-threatening complication of RNV, remains a major priority. In this study, we performed the an in-depth transcriptional and single-cell protein analysis of diabetic RNV tissue samples in order to examine the complex cellular and molecular interactions in an unbiased manner and to identify new therapeutic avenues. We show that RNV are characterized by an accumulation of endothelial cells, macrophages and myofibroblasts, and by an abundance of secreted ECM proteins such as SPARC, FN1 and several types of collagen. Specifically, we postulate that vitreous macrophages, also known as hyalocytes, have the ability to differentiate into α-SMA^+^ myofibroblasts suggesting a critical role for hyalocytes in the formation and contractile nature of RNV in PDR.

Our current knowledge of the pathophysiology of RNV rests mainly upon immunohistochemical and molecular studies on human samples (2, 11, 12) and preclinical animal models (44). For technical reasons, most previous studies had to focus on preselected and predefined cells and molecular pathways and were therefore limited in their power, as they could not comprise the multifactorial complexity of the disease. Furthermore, available diabetic animal models primarily mimic aspects of non-proliferative diabetic retinopathy and rarely exhibit retinal neovascularization, limiting their utility for PDR research. To address this gap in knowledge, the present study provides the first unbiased and comprehensive cellular and transcriptional analysis of human RNV membranes collected during vitreoretinal surgery.

Our sequencing analysis of RNV and ILM tissue revealed 3365 differentially upregulated factors in RNV. These DEG mainly contributed to GO biological processes, such as “*angiogenesis*” and diverse immune processes, e.g. “*leukocyte migration*”, “*adaptive immune response*” and “*complement activation*”, implicating an important role of both the innate and adaptive immune system in PDR. In addition, a plethora of ECM components, such as *SPARC*, *FN1* and various collagens, emerged among the most highly expressed genes in diabetic RNV. The conducted immunohistochemical analysis confirmed an enhanced FN1 and COL1 protein expression in RNV, which are important ECM components associated with the thickening of the capillary vascular basement membrane in diabetic retinopathy (45). The most highly expressed DEG in RNV, *SPARC*, appeared to be of particular interest, as it has previously been implied in the development of PDR by reports revealing increased SPARC protein levels in the vitreous of PDR patients (46). Preclinical evidence suggests that SPARC deletion has stimulating effects on ischemic retinopathy, RNV (47) and tumor angiogenesis by enhancing VEGF and MMP9 expression (48–50). Of note, the vascular endothelial growth factor (*VEGF*), which is currently the only clinically assessed PDR treatment option, did not emerge among the DEG in our RNA sequencing analysis of RNV. However, our analysis demonstrates an accumulation of both *SPARC* and the potent angiogenic molecule *MMP9* in RNV membranes supporting previous reports (51). MMP9 is a significant factor of intersection between angiogenesis and inflammatory response expressed in diabetic neovascular membranes (51) and assumed as an important molecule facilitating the disintegration of the blood-retina barrier observed in DR (52). Furthermore, MMP9 is capable of stimulating the expression of pro-inflammatory mediators and increasing the invasiveness of immune cells by cleaving major components of the basement membrane (52).

The cell type enrichment and proteomic analyses in this study identified endothelial cells, astrocytes and fibroblasts among the nine most enriched cell types in RNV, which is in line with previous reports (11). According to our high-dimensional single-cell IMC data different kinds of HLA-DR^+^ antigen-presenting cell types ranked among the most represented populations in RNV. In addition, we observed CD8^+^ and CD4^+^ lymphocytes in human RNV, in support of the prevailing notion of an important role of both innate and adaptive immunity in active PDR (53). Interestingly, among macrophages, anti-inflammatory CD206^+^ M2 macrophages in particular, which are thought to play an important role in debris clearance, wound healing, and angiogenesis, were increased in RNV (54). This finding is in accordance with earlier immunohistochemical studies reporting on CD163^+^ M2 macrophages in fibrovascular membranes of PDR patients and an absence of the M1 marker CD80 (55). In addition, M2 macrophages have been found to express significantly higher levels of SDF-1 (stromal cell-derived factor 1) and VEGF than M1 macrophages in a non-diabetic animal model of RNV, hereby regulating recruitment and differentiation of bone marrow-derived cells and further exacerbating RNV formation (56). The observation of M2 macrophages in fibrovascular membranes inevitably prompts questions about the origin of these cells and their functions in the disease process. Whether tissue-derived macrophages, such as retinal microglia and vitreal hyalocytes, or infiltrating monocyte-derived macrophages accumulate at RNV sites and modulate their formation is the subject of much debate (57). We have recently shown that tissue-resident retinal microglia accumulate at sites of experimental murine RNV and exceed by far the number of infiltrating macrophages from blood (15, 40). The close proximity of vitreous hyalcocytes located within the posterior vitreous cortex and RNV growing along its boundary makes it conceivable that at least some of the observed HLA-DR^+^ macrophages in RNV are vitreous hyalcocytes. The present study shows that numerous HLA-DR^+^ macrophages in RNV co-express α-SMA, strongly suggesting that macrophages contribute to the myofibroblast population in RNV. This notion is in line with previous findings demonstrating that human macrophages transdifferentiate into myofibroblasts in renal fibrosis (58). This hypothesis prompted us to assess the capacity of vitreal hyalocytes to transdifferentiate into α-SMA^+^ cells *in vitro*. Staining of FACS-enriched cultivated human vitreal hyalocytes revealed a strong expression of α-SMA *in vitro*. This suggests that human hyalocytes can transdifferentiate to myofibroblasts and may thus represent a component of the contractile fibrovascular membrane in advanced PDR. The concept of a contractile character of hyalocytes has been postulated previously based on studies on cultured bovine vitreous in the presence of PDGF-BB and TGF-β1 (10). In particular TGF-β, which was also significantly upregulated in human RNV in this study, can induce α-SMA expression in myeloid cells *in vitro* (59) and stimulates the contraction of hyalocytes *in vitro* (60). Taken together, these studies highlight the importance of M2 macrophages in the development of RNV and imply an important role for hyalocytes contributing to the myofibroblast pool. Thus, our data suggest that pharmacologic immunomodulation may be a potential therapeutic option to inhibit the formation of contractile fibrovascular membranes in PDR.

The screening for drug discovery based on the RNA sequencing data revealed verteporfin and imatinib as the most relevant potential pharmacologic treatment options for severe PDR. Imatinib is a 2-phenyl amino pyrimidine derivate, which acts as a specific inhibitor of tyrosine kinase enzymes and is used in leukemia treatment. A role of imatinib has been reported in suppressing M2-like polarization, which plays a decisive role in metastasis of lung cancer cells (61), and, according to our current findings, in the pathogenesis of RNV. Imatinib has already been shown to suppress retinal neovascularization in a mouse model of retinal neovascularization by targeting the platelet-derived growth factor (PDGF) without retinal side effects (62). PDGF is further involved in the contractile nature of bovine hyalocytes as reported previously (10). According to our analysis, an imatinib-induced suppression of RNV may attenuate both angiogenic and contractile tissue properties.

We acknowledge that our study has several limitations including the fact that control samples were obtained from patients with vitreoretinal disease, namely macular pucker (MP) or macular hole (MH). Nevertheless, both conditions are diseases of the vitreoretinal interface associated with posterior vitreous detachment and represent the most physiological conditions accessible in the clinical routine. We have previously shown that removed epiretinal tissue from MP and MH patients and hyalocytes collected in vitrectomy bags containing vitreous bodies are similar on a transcriptional level irrespective the underlying vitreoretinal pathology, indicating that the disease itself does not affect RNA expression patterns of hyalocytes, the only cells present in the healthy vitreous (9). The age difference between PDR and control patients in this study also resembles a relevant limiting factor. Since both MP and MH represent degenerative vitreoretinal interface diseases, thus occurring primarily in elderly patients, and severe PDR cases frequently develop in younger patients, the different age groups are not surprising, could however confound our results. Finally, the limited compilation of factors available for analysis within the xCell enrichment tool comprises another issue. The respective results presented in this work thus reflect only in xCell-available cell populations, while leaving out other cells types relevant in the pathogenesis of PDR, such as Müller cells.

Taken together, the transcriptional and single-cell protein characterization of RNV tissue in this study reveals an abundance of endothelial cells, immune cells and myofibroblasts, contributing to the progression of severe PDR by the expression of a variety of pro-angiogenic and pro-fibrotic factors. Our data implies an important role of transdifferentiating hyalocytes in the pathogenesis of diabetic vitreoretinal disease and suggests their modulation as a novel possible clinical approach.

## Data Availability Statement

The datasets presented in this study can be found in online repositories. The names of the repository and accession number can be found in the article.

## Ethics Statement

The studies involving human participants were reviewed and approved by Ethics Committee Freiburg. The patients/participants provided their written informed consent to participate in this study.

## Author Contributions

SB, JW, and ASch conducted experiments and analyzed data. RH, GP, HS, SK, YL, and MB performed experiments. HF, AL, FB, ASt, HA, and CL performed surgical procedures. BB, DB, HA, GS, and CL contributed to design of the study and interpretation of the data. All authors contributed to the article and approved the submitted version.

## Funding

This study was conducted with the generous support of the Dr. Werner Jackstädt Foundation and the Grimmke Foundation. SB is a Clinician Scientist Fellow of the Berta Ottenstein Program, Faculty of Medicine, University of Freiburg, Germany.

## Conflict of Interest

The authors declare that the research was conducted in the absence of any commercial or financial relationships that could be construed as a potential conflict of interest.

## Publisher’s Note

All claims expressed in this article are solely those of the authors and do not necessarily represent those of their affiliated organizations, or those of the publisher, the editors and the reviewers. Any product that may be evaluated in this article, or claim that may be made by its manufacturer, is not guaranteed or endorsed by the publisher.

## Supplementary Material

Supplementary Figure 1 Gene ontology analysis of retinal neovascularization (RNV). **(A)** Readplot of differentially expressed genes in RNV; factors associated with the GO term “angiogenesis” are highlighted (upregulated genes in magenta, downregulated genes in blue, not differentially expressed genes in grey, top expressed angiogenesis-associated genes in green; the most strongly upregulated genes are labeled). **(B)** Readplot of differentially expressed genes in RNV; factors associated with the GO term “immune response” are highlighted (upregulated genes in magenta, downregulated genes in blue, not differentially expressed genes in grey, immune response-associated genes in green; the most strongly upregulated genes are labeled). **(C)** Top five most highly expressed transcripts in the three most enriched GO terms from 3C. **(D)** Most significant Gene ontology (GO) molecular functions clusters of the significantly upregulated mRNA transcripts in RNV. Color coding of the dots according to the adjusted *p* value, size of the dots according to the count of transcripts associated with the respective GO term. GAG, glycosaminoglycan. IG, immunoglobulin. PK, protein kinase. TGF-β, transforming growth factor beta. PDGF, platelet-derived growth factor. **(E)** Cnetplot of the top expressed genes in the most disease-relevant GO molecular functions. Color coding of the transcripts according to the log2 fold change of mean expression.

Supplementary Figure 2 Negative and autofluorescence controls for immunohistochemistry. **(A)** Negative controls, omitting the primary antibodies, for CD206 (Cluster of Differentiation 206), FN1 (Fibronectin) and SPARC (secreted protein acidic and rich in cysteine) immunohistochemical stainings (shown in **Figures 2I** and **3E**) in retinal neovascularization (RNV) and internal limiting membrane (ILM) tissue samples. DAR 568, donkey anti-goat 568. DAM 488, donkey anti-mouse Alexa Fluor 488. DAR Cy5, donkey anti-rabbit Cy5. **(B)** Autofluorescence controls for CD206, FN1 and SPARC immunohistochemical stainings in RNV and ILM (shown in **Figures 2I** and **3E**). Nuclei are counterstained with DAPI (4′,6-Diamidin-2-phenylindol). Scale bars correspond to 100 μm.

Supplementary Figure 3 Imaging Mass Cytometry on retinal neovascularization and epiretinal membranes: representative sections of all plausible stainings.

Supplementary Figure 4 Immunohistochemical staining of plated hyalocytes from a PDR patient. IBA-1 (green), α-SMA (yellow) and Phalloidin (red). Nuclei are counterstained with DAPI (4′,6-Diamidin-2-phenylindol). Scale bars correspond to 25 μm.
