# Supplementary figures and images for: In-Depth Molecular Characterization of Neovascular Membranes Suggests a Role for Hyalocyte-to-Myofibroblast Transdifferentiation in Proliferative Diabetic Retinopathy
Source: Front Immunol. 2021 Nov 2;12:757607. doi: 10.3389/fimmu.2021.757607 (PMC8593213; doi:10.3389/fimmu.2021.757607)

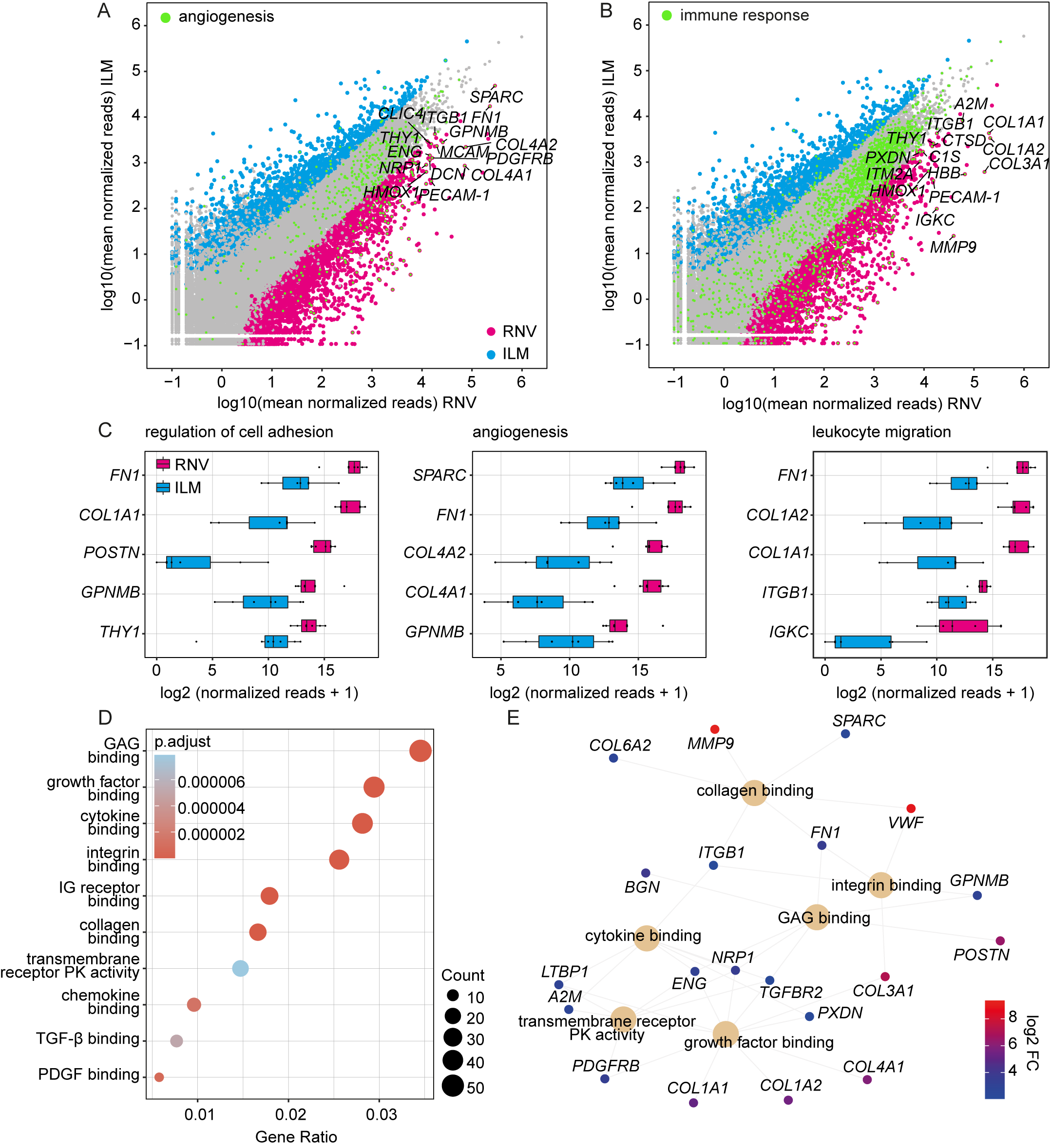

Supplement: Supplementary Figure 1 — Gene ontology analysis of retinal neovascularization (RNV). (A) Readplot of differentially expressed genes in RNV; factors associated with the GO term “angiogenesis” are highlighted (upregulated genes in magenta, downregulated genes in blue, not differentially expressed genes in grey, top expressed angiogenesis-associated genes in green; the most strongly upregulated genes are labeled). (B) Readplot of differentially expressed genes in RNV; factors associated with the GO term “immune response” are highlighted (upregulated genes in magenta, downregulated genes in blue, not differentially expressed genes in grey, immune response-associated genes in green; the most strongly upregulated genes are labeled). (C) Top five most highly expressed transcripts in the three most enriched GO terms from 3C. (D) Most significant Gene ontology (GO) molecular functions clusters of the significantly upregulated mRNA transcripts in RNV. Color coding of the dots according to the adjusted p value, size of the dots according to the count of transcripts associated with the respective GO term. GAG, glycosaminoglycan. IG, immunoglobulin. PK, protein kinase. TGF-β, transforming growth factor beta. PDGF, platelet-derived growth factor. (E) Cnetplot of the top expressed genes in the most disease-relevant GO molecular functions. Color coding of the transcripts according to the log2 fold change of mean expression. [file Image_1.tif]

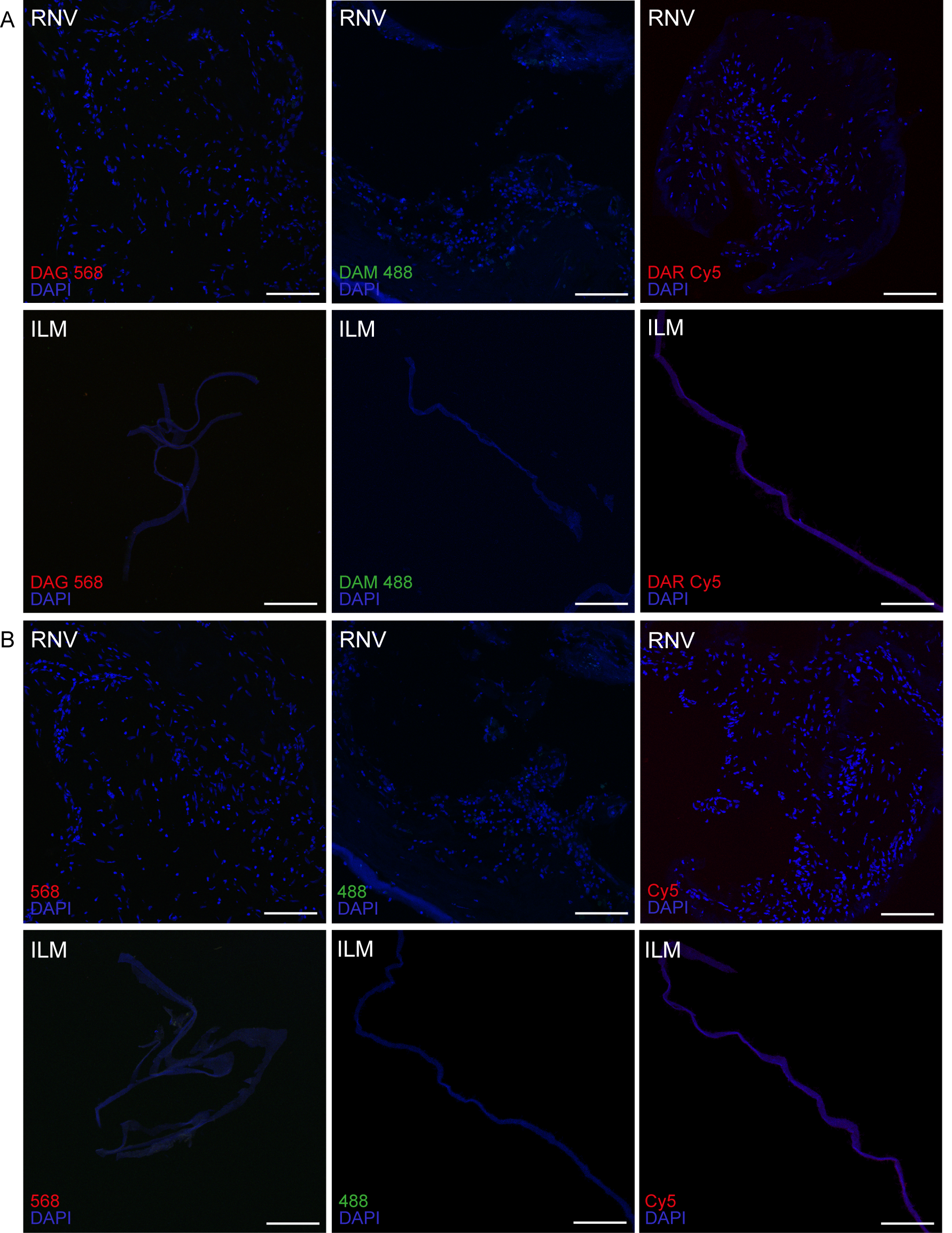

Supplement: Supplementary Figure 2 — Negative and autofluorescence controls for immunohistochemistry. (A) Negative controls, omitting the primary antibodies, for CD206 (Cluster of Differentiation 206), FN1 (Fibronectin) and SPARC (secreted protein acidic and rich in cysteine) immunohistochemical stainings (shown in Figures 2I and 3E ) in retinal neovascularization (RNV) and internal limiting membrane (ILM) tissue samples. DAR 568, donkey anti-goat 568. DAM 488, donkey anti-mouse Alexa Fluor 488. DAR Cy5, donkey anti-rabbit Cy5. (B) Autofluorescence controls for CD206, FN1 and SPARC immunohistochemical stainings in RNV and ILM (shown in Figures 2I and 3E ). Nuclei are counterstained with DAPI (4′,6-Diamidin-2-phenylindol). Scale bars correspond to 100 μm. [file Image_2.tif]

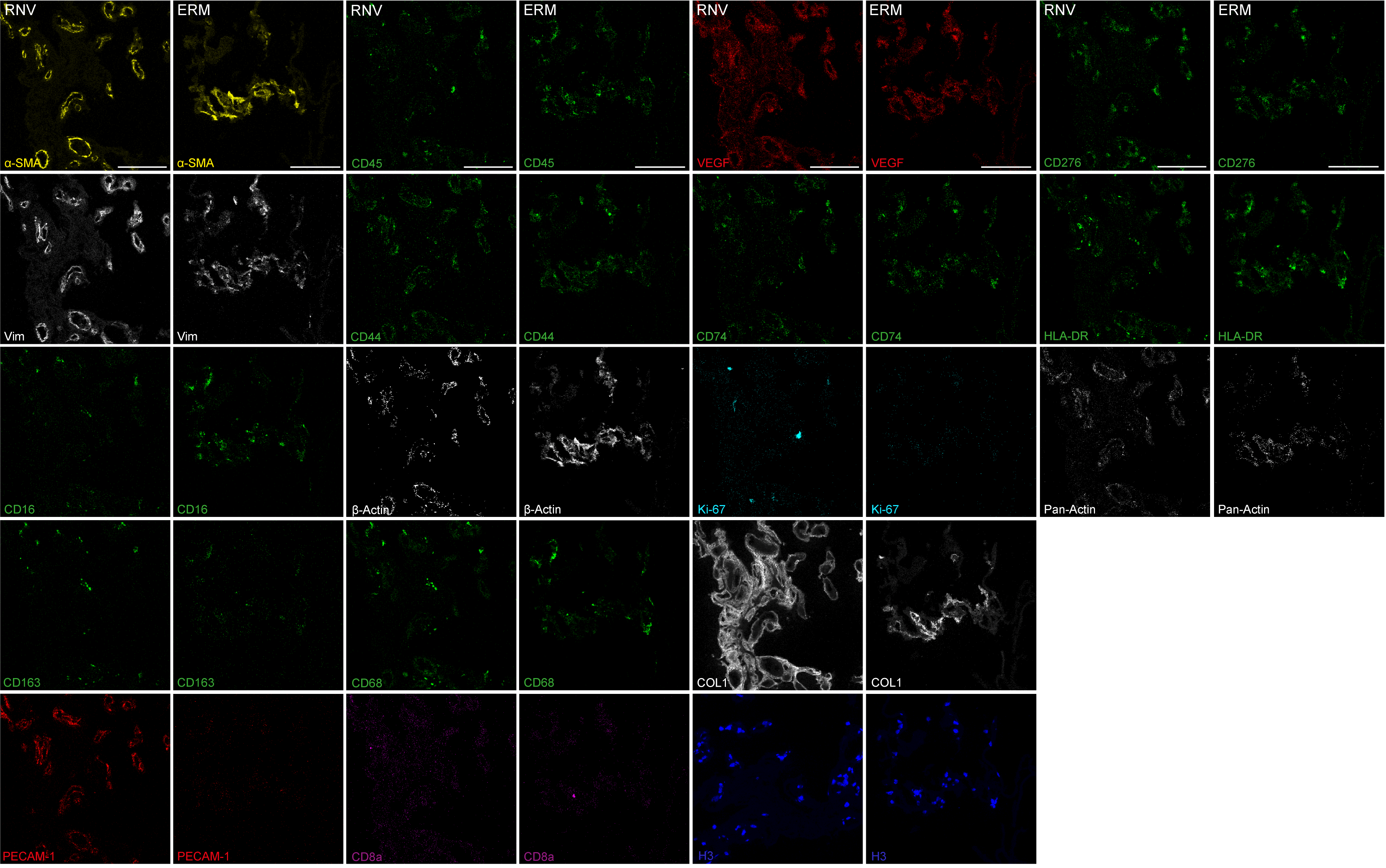

Supplement: Supplementary Figure 3 — Imaging Mass Cytometry on retinal neovascularization and epiretinal membranes: representative sections of all plausible stainings. [file Image_3.tif]

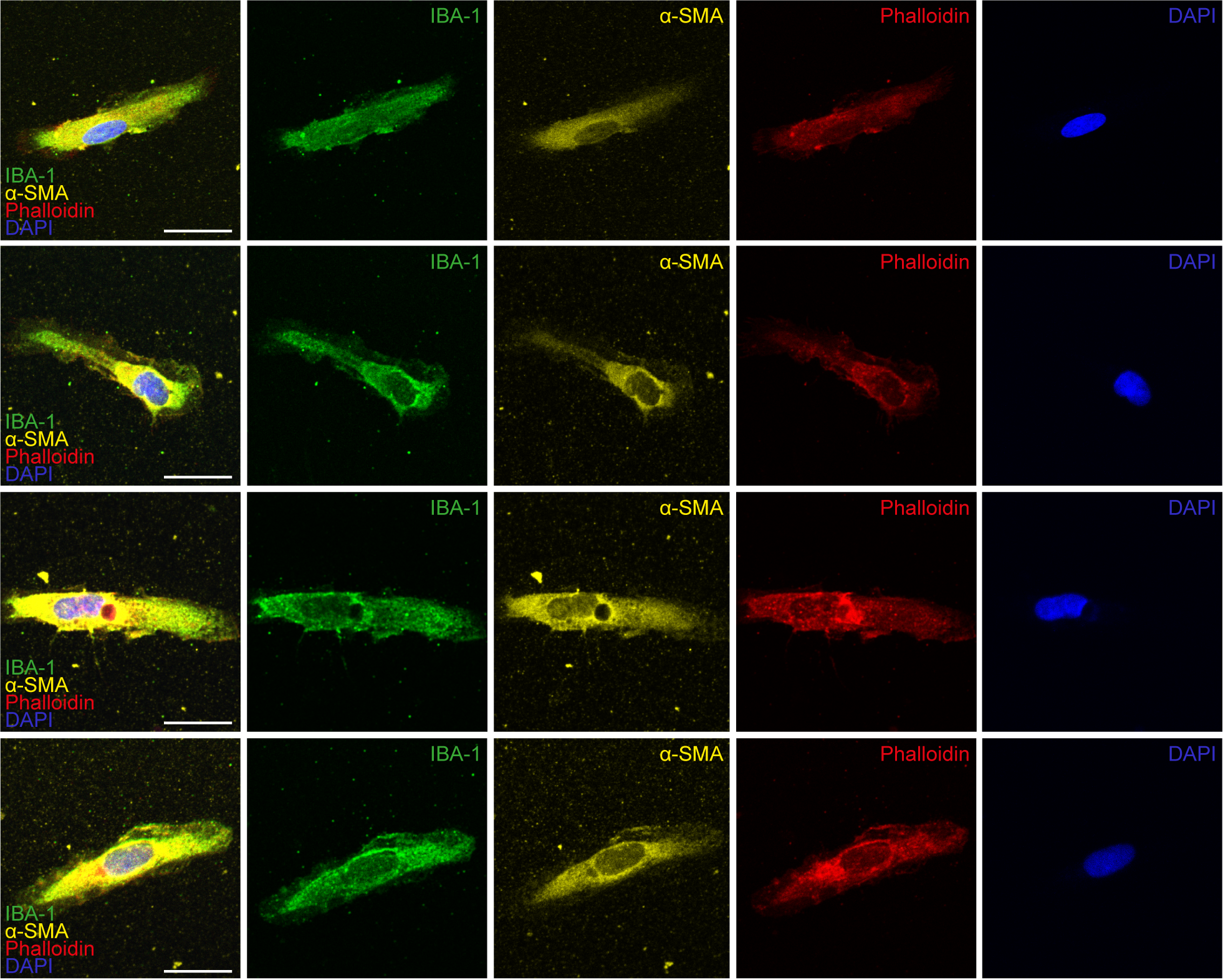

Supplement: Supplementary Figure 4 — Immunohistochemical staining of plated hyalocytes from a PDR patient. IBA-1 (green), α-SMA (yellow) and Phalloidin (red). Nuclei are counterstained with DAPI (4′,6-Diamidin-2-phenylindol). Scale bars correspond to 25 μm. [file Image_4.tif]
