# Supplementary material for: In-Depth Molecular Characterization of Neovascular Membranes Suggests a Role for Hyalocyte-to-Myofibroblast Transdifferentiation in Proliferative Diabetic Retinopathy
Source: Front Immunol. 2021 Nov 2;12:757607. doi: 10.3389/fimmu.2021.757607 (PMC8593213; doi:10.3389/fimmu.2021.757607)
Supplement: Supplementary file 5 [file Table_1.pdf]

Supplementary Table 1. Patients' characteristics.

#	Age (years)	Sex	DM Type	Ocular Diagnosis	Lens status	Previous vitrectomy	Previous anti-VEGF treatment	Previous PRP treatment	Readout
1	40	M	I	PDR	phakic	no	no	yes	RNA-Seq
2	35	M	I	PDR	phakic	no	no	yes	RNA-Seq
3	29	M	I	PDR	phakic	yes	> 3 months	yes	RNA-Seq
4	29	F	I	PDR	phakic	no	no	no	RNA-Seq
5	56	M	II	PDR	phakic	no	no	no	RNA-Seq
6	20	F	I	PDR	phakic	no	no	yes	RNA-Seq
7	60	M	I	PDR	pseudophakic	no	> 3 months	yes	RNA-Seq
8	32	F	I	PDR	phakic	no	no	yes	IHC
9	29	F	I	PDR	phakic	no	4 days prior to surgery	yes	IHC
10	29	M	I	PDR	pseudophakic	yes	no	yes	IMC
11	58	F	II	PDR	phakic	no	no	yes	IMC
12	45	F	II	PDR	phakic	no	no	no	IMC
13	52	F	II	PDR	phakic	no	no	yes	IMC
14	33	M	I	PDR	phakic	no	> 3 months	yes	IMC
15	86	M	II	PDR	pseudophakic	no	no	yes	Cell culture
16	52	F	I	PDR	phakic	no	no	yes	Cell culture
17	37	M	I	PDR	phakic	no	> 3 months	yes	Cell culture
18	73	M	II	PDR	phakic	no	> 3 months	yes	Cell culture
19	40	F	II	PDR	phakic	no	> 3 months	yes	Cell culture
20	71	M	-	MP	phakic	no	no	no	RNA-Seq
21	83	M	-	MP	pseudophakic	no	no	no	RNA-Seq
22	79	M	-	MP	phakic	no	no	no	RNA-Seq
23	66	F	-	MP	phakic	no	no	no	RNA-Seq
24	72	F	-	MP	phakic	no	no	no	RNA-Seq
25	62	M	-	MP	pseudophakic	no	no	no	RNA-Seq
26	62	M	-	MP	phakic	no	no	no	RNA-Seq
27	75	M	-	MP	phakic	no	no	no	RNA-Seq
28	70	F	-	MP	pseudophakic	no	no	no	RNA-Seq
29	69	M	-	MP	phakic	no	no	no	RNA-Seq
30	77	M	-	MP	pseudophakic	no	no	no	IHC
31	77	M	-	MP	phakic	no	no	no	IHC
32	64	F	-	MP	phakic	no	no	no	IMC
33	68	M	-	MP	phakic	no	no	no	IMC
34	74	M	-	MP	pseudophakic	no	no	no	IMC
35	65	F	-	MH	phakic	no	no	no	RNA-Seq
36	69	M	-	MH	phakic	no	no	no	RNA-Seq
37	79	F	-	MH	pseudophakic	no	no	no	RNA-Seq
38	67	F	-	MH	phakic	no	no	no	RNA-Seq
39	67	M	-	MH	phakic	no	no	no	RNA-Seq
40	61	F	-	MH	phakic	no	no	no	RNA-Seq
41	66	F	II	MH	pseudophakic	no	no	no	RNA-Seq
42	80	F	-	MH	pseudophakic	no	no	no	IHC
43	77	F	-	MH	pseudophakic	no	no	no	IHC

DM, diabetes mellitus. VEGF, vascular endothelial growth factor. PRP, panretinal photocoagulation. PDR, proliferative diabetic retinopathy. MP, macular pucker. MH, macular hole. IHC, immunohistochemistry. IMC, Imaging Mass Cytometry.
