# Supplementary material for: In-Depth Molecular Characterization of Neovascular Membranes Suggests a Role for Hyalocyte-to-Myofibroblast Transdifferentiation in Proliferative Diabetic Retinopathy
Source: Front Immunol. 2021 Nov 2;12:757607. doi: 10.3389/fimmu.2021.757607 (PMC8593213; doi:10.3389/fimmu.2021.757607)
Supplement: Supplementary file 6 [file Table_2.pdf]

Supplementary Table 2. Antibodies, respective clones and conjugated metals for Imaging Mass Cytometry Analysis.

Target	Clone	Metal
α-SMA	1A4	141Pr
EGFR	D38B1	142Nd
Vimentin	D21H3	143 Nd
CD16	EPR16784	146 Nd
CD163	EDHu-1	147Sm
Pan-Keratin	C11	148Nd
CD11b	EPR1344	149Sm
CD274 (PD-L1)	SP142	150Nd
CD31 (PECAM-1)	EPR3094	151Eu
CD45	D9M8I	152Sm
CD44	IM7	153Eu
β-Actin	2F1-1	154Sm
E-Cadherin	2,40E+11	158Gd
CD68	KP1	159Tb
CD8a	C8/144B	162Dy
VEGF	G153-694	163Dy
Arginase 1	D4E3M	164Dy
CD74	LN2	166Er
Granzyme B	EPR20129-217	167Er
Ki-67	B56	168Er
Collagen type I	Polyclonal	169Tm
Histone H3	D1H2	171Yb
CD276 (B7-H3)	Polyclonal	173Yb
HLA-DR	LN3	174Yb
Pan-Actin	D18C11	175Lu
Nucleic Acid		191Ir/193Ir
